# A shape-changing haptic navigation interface for vision impairment

**DOI:** 10.1038/s41598-024-79845-7

**Published:** 2024-12-10

**Authors:** Robert Quinn, Stephen Murtough, Henry de Winton, Brandon Ellis-Frew, Sebastiano Zane, Jonathan De Sousa, Theofilos Kempapidis, Renata S. M. Gomes, Adam J. Spiers

**Affiliations:** 1 MakeSense Technology Ltd, London, UK; 2 Manipulation and Touch Lab, Department of Electrical and Electronic Engineering, Imperial College London, London, UK; 3 BRAVO VICTOR, London, UK; 4 Northern Hub for Veterans and Military Families Research, Northumbria University, Newcastle Upon Tyne, UK

**Keywords:** Eye diseases, Electrical and electronic engineering, Mechanical engineering

## Abstract

Individuals with visual impairment (VI) require aids such as white canes and guide dogs to navigate their environments. Modern localisation technologies have the capacity to transform the way individuals with VI navigate surroundings, but they have yet to do so. A critical barrier is the inability of human–machine interfaces to communicate precise navigation instructions non-visually. We present a shape changing haptic interface (Shape) that provides spatial guidance in two dimensions via bending of its body. Individuals with VI and sighted individuals were recruited to locate virtual targets in 3D space using Shape and vibration feedback (Vibration), and sighted individuals were also asked to visually locate targets. Throughout, device orientation and position were tracked in real-time using a virtual reality system. Individuals with VI located targets significantly faster and more efficiently using Shape, than with Vibration, and there were no significant differences in time or efficiency between Shape and natural vision. Moreover, participants scored Shape significantly more positively than Vibration in a Likert user experience survey, while no significant differences were observed between Shape and natural vision. Here, we provide compelling evidence for the application of a new shape-changing haptic interface as part of an effective future digital navigation system for individuals with VI.

## Introduction

Individuals with vision impairment (VI) rely on navigation aids to safely avoid obstacles and travel to destinations without the help of another person^1^. Two established and commonly used navigation aids are white canes and guide dogs^2^. While both are proven and well used, they are limited in several ways. The traditional white cane is an exclusionary guidance tool, i.e. it tells its user where *not* to go. Through physical contact with local surroundings (and the resulting mechanotactile haptic feedback to the hand) a person can find gaps or obstacles and thus determine safe routes through a process of elimination. Guide Dogs work very differently. They offer their owner active guidance through a range of feedback mechanisms, though largely rely on mechanotactile haptic guidance via their harness handle^3^. A guide dog can effectively determine its owner’s intentions and guide them along safe routes, but the user remains ultimately responsible for determining their destination^4^. Moreover, while a guide dog can dramatically improve the mobility and confidence of an individual with VI^5^, some do not want to keep a dog, and it can be frustrating if the dog becomes distracted or displays undesirable behaviour^6–8^. In addition, guide dogs are in limited supply due to the expensive and complex training required, costing approximately £15 k per year per animal^9^. While Guide Dogs UK have the capacity to service 4000 guide dogs at any one time, there are 300,000 severely VI persons in the UK^9,10^.

In recent years, the technology required to localise a person in space with centimetre-level precision has matured, with advances such as Simultaneous Localisation and Mapping (SLAM), computer vision, and highly accurate GPS^11^. In principle, these technologies could deliver similar benefits to individuals with VI as guide dogs do; however, they have not found widespread use within the vision-impaired community. The inability of current non-visual interfaces to communicate enough navigational information has limited this impact.

Auditory interfaces have long been investigated to provide navigational guidance to VI persons. Natural language audio cues are effective for providing high-level guidance, such as ‘turn left at the next corner’. However, this type of guidance can be ambiguous and often confusing^12^. Pitch and tone based auditory guidance systems can provide more generalised and responsive instruction but require considerable training to use. Furthermore, previous work has found audio guidance can inhibit a VI person’s capacity to perceive and appreciate their environment and can prevent them from hearing potentially important warning sounds of imminent hazards^13–16^.

Alternatively, directions and warning signals can be provided by haptic feedback in the form of vibrational stimuli^17–23^. While these are effective at alerting a user to an event, such as a message on a smartphone, they can quickly become irritating and distracting and can also lead to numbness following prolonged periods of usage^24–28^. Indeed, these limitations suggest a need for an alternative form of interface that is appropriate for digital navigation devices for VI persons.

A promising option is to provide spatial information in the form of shape-changing haptic feedback. It has been shown that human hands are inherently adept at perceiving shape change with relatively low cognitive burden^29^, and we have demonstrated the potential of this interface through multiple studies of a variety of device prototypes^3,4^. Most recently, we tested the S-BAN, a shape-changing haptic device that outperformed previously published designs^4^. In a navigation study, the S-BAN was highly effective at guiding sighted participants to virtual targets, and it was found that during the study user head pose was more focused on the environment than the device, relative to a visual tool (a smartphone proxy), which may have important safety benefits for use in an outside real-world environment^4^.

**Fig. 1:**
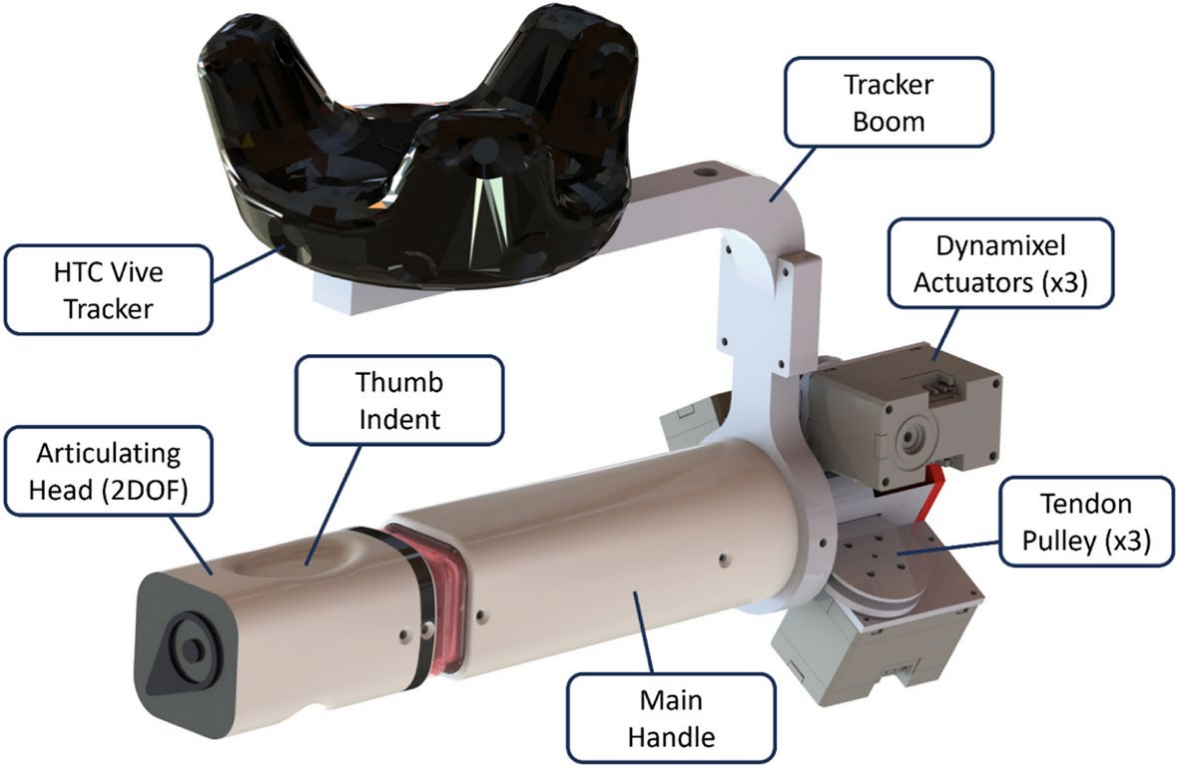
A summary of the Shape haptic device. The Shape haptic device comprises a main handle attached to an articulating head that can bend with 2 degrees of freedom (pitch and yaw). Participants were intended to hold the device by the main handle, and to place their thumb on the thumb indent groove on top of the articulating head, which provides the user with haptic feedback as and when the head bends to navigate to a target. 3 × Dynamixel actuators and 3 × tendon pulleys were used to power and control the motion of the device. A linear resonant actuator was contained in the handle, which provided a vibration sensation. A boom was attached to the base of the device so that the HTC Vive tracker could be positioned above without interfering with Shape’s capacity to provide navigational haptic feedback to a participant.

In this paper, we build on our previous work and present a new shape-changing haptic device specifically designed for VI navigation. Our device, which we describe throughout as Shape, is handheld and able to bend in the user’s hand via 2 degrees of freedom (DOF) to represent direction. Its design is described in Fig. 1. Briefly, Shape locks-on to targets/directions in 3D space, which we refer to as vectors; Shape then physically bends along the target vector while held in the hand; and sensing the bending direction through haptic feedback (i.e. touch), the user follows the curvature to point Shape at the intended target/direction. By *pointing*, the user ‘feels’ target directions via *proprioception*, and the device straightens as it is pointed towards the target; this gesture is found to be well understood even by congenitally blind children^30^. Moreover, Shape continuously updates at a rate of 50 Hz, responding to user/target movements. This design generalises the approach taken with the S-BAN work to communicate any vector in 3D^4^, which is necessary for precise local navigation tasks that include an altitude component, such as finding a door handle.

**Fig. 2:**
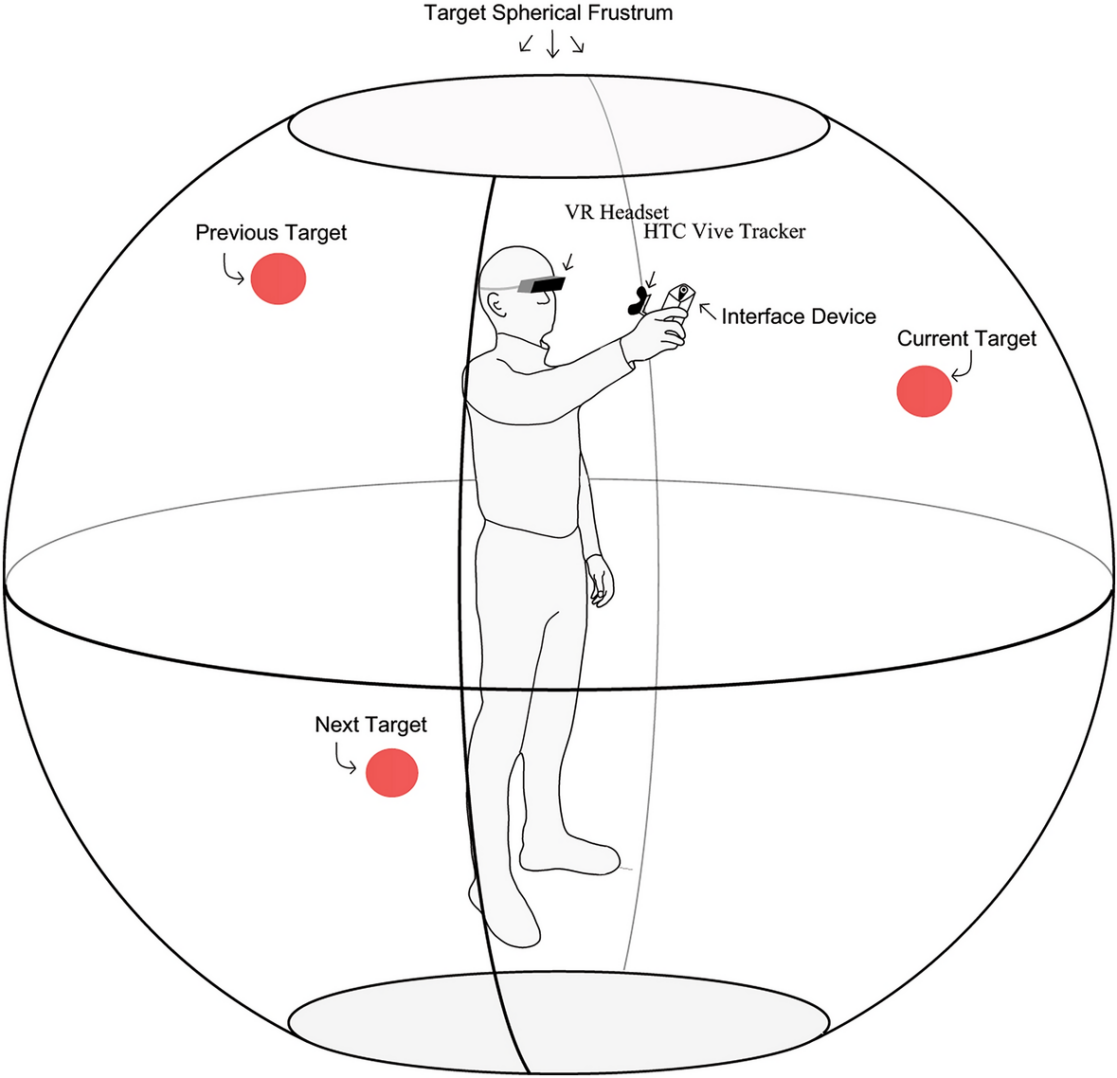
A summary of the experiment design. Schematic depicting the experimental design that was followed to test Shape and Vibration interface device types. Participants were instructed to stand within the test space, and a 3m radius spherical frustrum (sphere is not to scale in the figure) was constructed in virtual space, around the participant, using a virtual reality system (HTC Vive tracker). 60 targets were generated sequentially on the sphere surface, and the device types were used to direct the participant to point at the target, within 7 degrees for 2 s. Once achieved, a new target would appear for the participant to point at. The same sequence sets were used for all participants and device types. At the beginning of each task the frustrum was recentred on the chest tracker. The starting zenith angle of each target from the next was normally distributed with a mean of 90 degrees. As the virtual targets are invisible, no participants were blindfolded.

We ultimately intend for Shape to be a comprehensive VI navigation device that is integrated with a computer vision system so that it can provide full guidance in real-world scenarios. However, in this study, we perform a first spatial guidance assessment of Shape in a controlled indoor situation. We recruited sighted and VI individuals to locate a series of 60 invisible virtual targets (which have no physical component in the study space) in 3D space using Shape and a 1-DOF vibrational device (termed Vibration), which represents the standard of digital navigational device technologies currently available. Sighted participants also located the virtual targets through sight, using a virtual reality headset. Participants do not wear a headset nor blindfold for the Shape or Vibration tasks. For all modes, the device had to be aimed at a virtual target with an accuracy below the 7-degree zenith angle threshold and held within that range for two seconds. At this point the next virtual target in the sequence was presented. Device orientation and position were recorded throughout using an HTC Vive virtual reality localisation system. The trial design is described in Fig. 2. We found that both VI and sighted participants located targets significantly faster and more efficiently using Shape, and users frequently scored Shape more positively than Vibration on qualitative measures. These results provide valuable insights into the future role that shape-changing haptic devices may play in navigation for people with VI, and we aim to use these findings as a springboard to refine and optimise our device for use in a real-world setting.

## Results

We recruited 10 participants with VI and 10 sighted participants to participate in this phase one trial (Table 1). The VI sample group were all legally blind by current UK definitions. They ranged from 26 to 85 years old, included 8 male and 2 female participants, and covered several eye conditions, including toxic myopia, retinitis pigmentosa, and age-related macular degeneration, as detailed in Table 1. The sighted sample group ranged from 20 to 30 years old, included 6 male and 4 female participants, and all participants were confirmed to have no vision issues or complaints. All participants completed full task sets for Shape and Vibration, and sighted participants completed extra Sighted task sets where they were asked to find the targets using natural vision. Informed consent was obtained from all participants.

**Table 1:** A summary of trial participant information.

Participant ID	Sex	Age (years)	Type of visual impairment
With visual impairment			
P1	M	65	Toxic myopia
P2	M	43	Macular degeneration and central vision loss
P3	M	67	Myopic choroidal neovascularisation
P4	F	70	Retinopathy of prematurity
P5	M	85	Age-related macular degeneration
P6	F	26	Retinitis pigmentosa, close to fully blind
P7	M	38	Right eye prosthetic, left eye cataract removed, and secondary glaucoma
P8	M	56	Tunnel vision due to bilateral occipital infarction brain injury
P9	M	53	Retinitis pigmentosa
P10	M	44	Leber congenital amaurosis
Sighted			
S1	M	30	
S2	M	26	
S3	F	27	
S4	M	26	
S5	M	25	
S6	F	29	
S7	M	20	
S8	F	25	
S9	F	24	
S10	M	27	

*M* Male, *F* Female.

### Assessing time taken to locate virtual targets

**Fig. 3:**
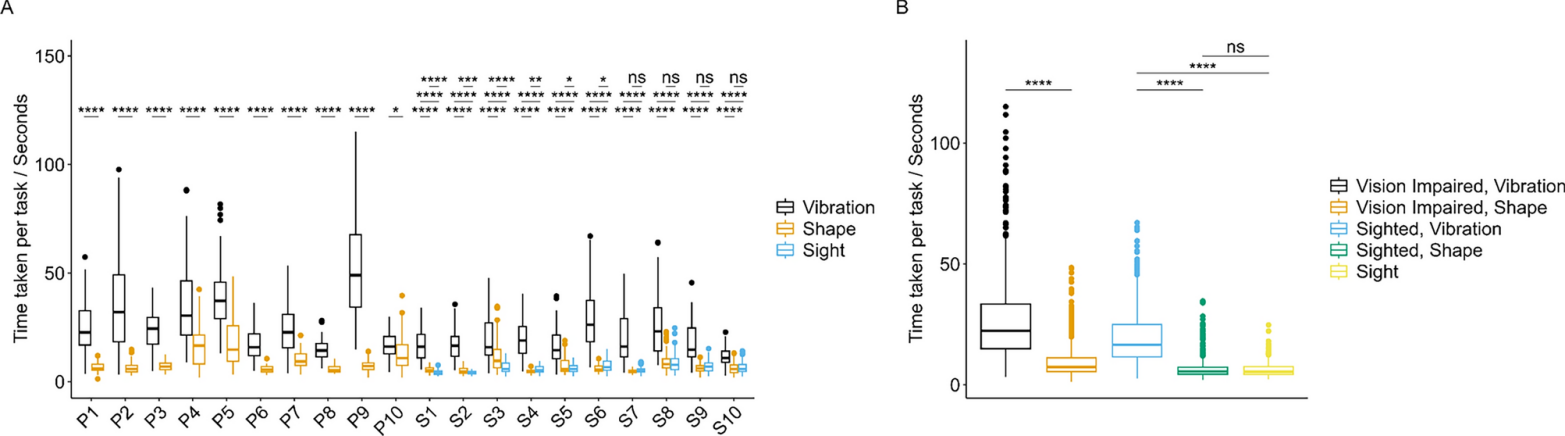
Time taken to complete tasks for participants and device types. Boxplots depicting time taken in seconds to complete tasks for (**A**) each participant for each device type (n = 20), and for (**B**) each device type, with participants grouped according to vision type (n = 10 participants with visual impairment; n = 10 sighted participants). Boxplots are colour coded as described in the plot legend. Two-sample t-tests were performed, comparing between groups, and Bonferroni correction was applied. Adjusted p-values are shown as significance stars; *ns* Not significant, *p < 0.05, **p < 0.01, ***p < 0.001, ****p < 0.0001. Plots produced using ggplot2 in R.

First, we assessed time taken to locate targets for each participant for each device type (Fig. 3). Targets deemed to include data glitches and/or to be incomplete were removed prior to plotting, and statistical outliers were removed using the interquartile range (IQR) method. All participants located targets significantly faster using Shape, relative to Vibration (Fig. 3A). Moreover, data for Vibration were widely spread and exhibited a large range, while data for Shape were frequently less variable about the median (Fig. 3A). In addition, there was no significant difference in time taken to locate targets between Shape and natural vision for the sighted participants, S7, S8, S9, and S10, highlighting the effectiveness of Shape for guiding and navigating individuals. When participants were grouped according to vision status (with VI or sighted), time taken to locate targets was significantly reduced for Shape, relative to Vibration, for both participants with VI and natural vision (Fig. 3B). Moreover, there was no significant difference in time taken to locate targets between Shape and natural vision for sighted participants (Fig. 3B); as above, highlighting the efficacy of the Shape device interface.

### Measuring how efficiently participants located virtual targets

Next, we designed a metric to measure how efficiently participants located targets. Device orientations were recorded in our data as quaternion values, and we used these to calculate the following metric. We defined the optimum path that a userf could take for a given task as the angular distance between starting and ending quaternion rotations, or rather, the angular distance between these rotations along the spherical linear interpolation path; and we defined the actual path taken by a participant as the cumulative angular distance between all recorded quaternion data points. Data were then processed to give an efficiency percentage (where, if followed, the optimum path would provide an efficiency readout of 100%).

**Fig. 4:**
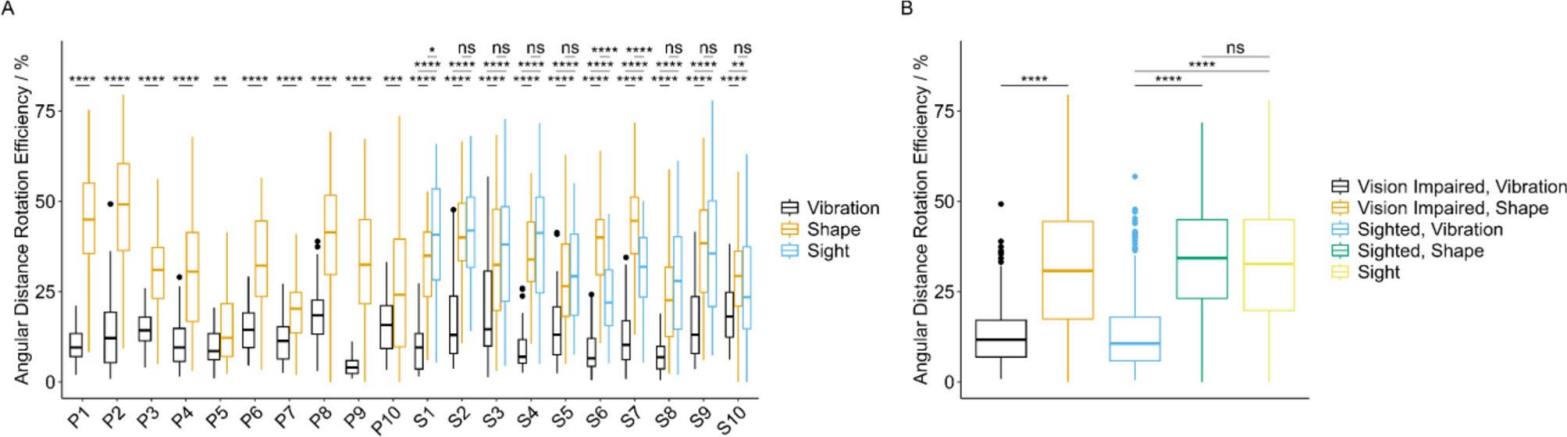
Angular distance rotation efficiency for participants and device types. Boxplots showing angular distance rotation efficiency for (**A**) individual participants (n = 20) and device types, and (**B**) participants grouped by vision type and device type (n = 10 participants with visual impairment; n = 10 sighted participants). Boxplots are colour coded as described in the plot legend. Two-sample t-tests were performed, comparing between groups, and Bonferroni correction was applied. Adjusted p-values are shown as significance stars; *ns* Not significant, *p < 0.05, **p < 0.01, ***p < 0.001, ****p < 0.0001. Plots produced using ggplot2 in R.

All participants located tasks significantly more efficiently using Shape, relative to Vibration (Fig. 4A). Moreover, for 7 sighted participants, there was statistically no significant difference in rotation efficiency between Shape and natural vision, underscoring the validity of Shape as a navigational guidance tool (Fig. 4A). When participants were grouped according to vision status (with VI or sighted), rotation efficiency was significantly elevated for Shape, relative to Vibration, for both sample groups (Fig. 4B). Moreover, as found for several participants in Fig. 4A, there was no significant difference in rotation efficiency between Shape and natural vision in the sighted participant sample group (Fig. 4B). Of note, these rotation efficiency data generally display a large spread and range for all device types for most participants. This confirms that for some targets (across all device types), participants performed poorly in locating the target; however, our data also suggests that on average, participants found targets more efficiently using the Shape device interface, and on several occasions, they did so comparably to using natural vision.

**Fig. 5:**
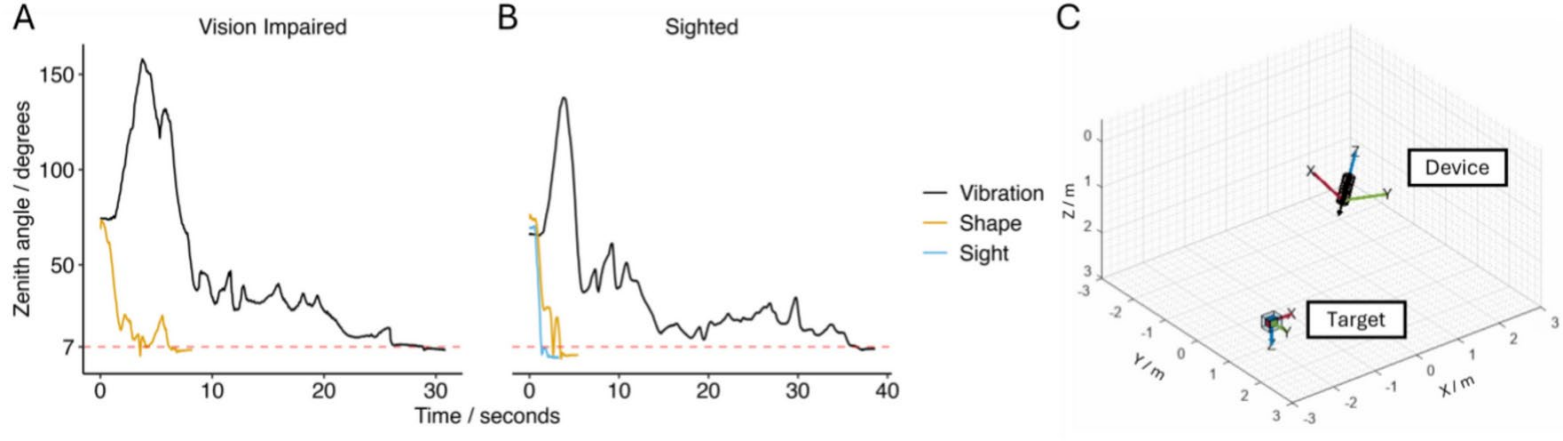
Representative targeting examples for all task conditions for VI and sighted participants. Line graphs showing zenith angle change (in degrees) over time (in seconds) for (**A**) Shape and Vibration for a single participant with VI (participant P6, target number 4), and (**B**) Shape, Vibration, and Sight for a single sighted participant (participant S4, target number 4). The dashed horizontal line at 7 degrees indicates the pre-defined error range for when a device is pointing at the target. When pointing at the target, the participant must keep within the 7-degree range for 2 s to complete the task. Plot produced using ggplot2 in R. Supplementary animations (visualised in MATLAB) illustrate device position and orientation relative to the (fixed) target for the cases shown in A and B. A single frame from one of these animations is displayed **(C)**. The forward direction of the device is represented as an arrow.

Representative examples for locating targets were generated for single participants with VI and sight and are illustrated in Fig. 5. P6 was chosen as a representative participant with VI, and target number 4 was visualised as the efficiency value for Vibration (15.3%) was close to the mean (14.4%). The same target was chosen and visualised for sighted participant S4. When using Shape, P6 located the target quickly and with a smooth and consistent path, however when using Vibration, P6 first pointed away from the target and then took a substantially longer time to locate the target (Fig. 5A). A similar pattern was observed for S4, who located the target quickly and smoothy with both Shape and natural vision but struggled to locate the target with Vibration (Fig. 5B). A 3D visualisation of this setup (for both participants) is shown in Fig. 5C. Animations for P6 locating target number 4 are shown in Supp. Fig. 1.

### Evaluating participant user experience for the different device interfaces

**Fig. 6:**
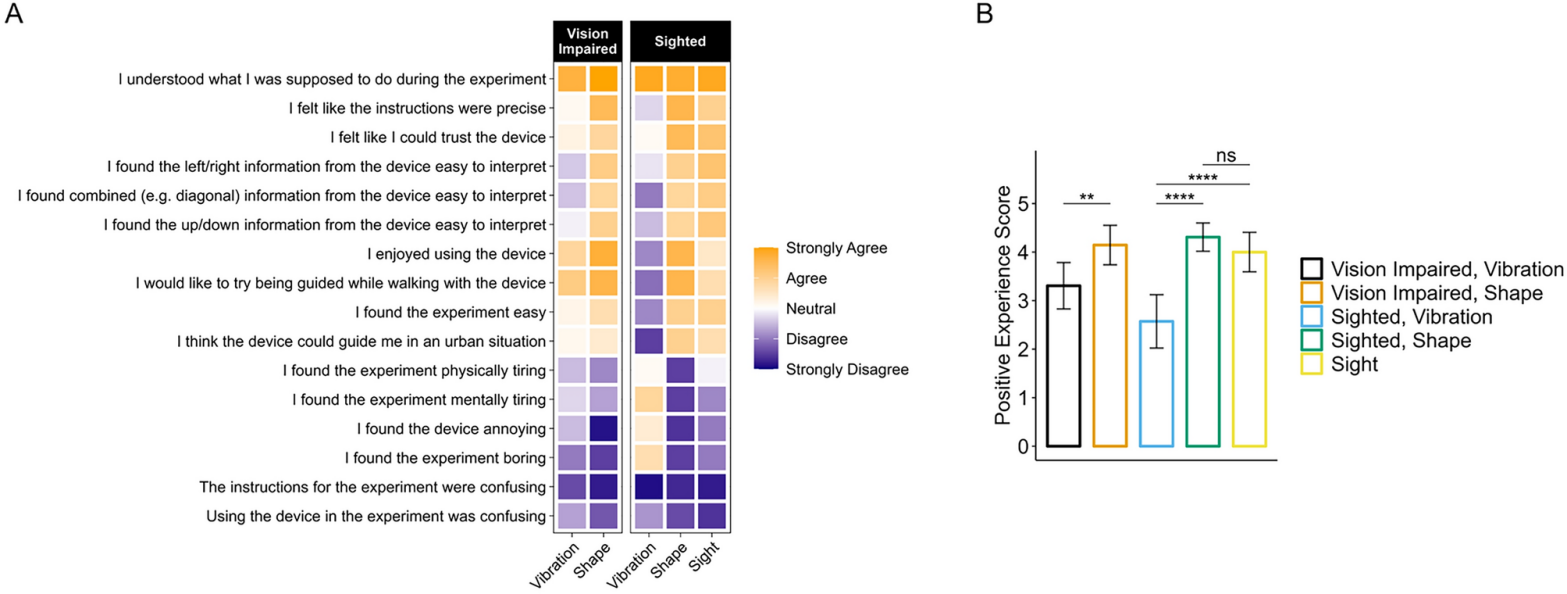
Mean Likert scores for participant user experience. (**A**) Heatmap depicting mean scores for Likert survey data. Data are grouped by vision type and device type (n = 10 participants with visual impairment; n = 10 sighted participants)., and a coloured gradient scale is shown indicating the average answer provided by each group, with pure orange representing strongly agree, pure white representing neutral, and pure purple representing strongly disagree. Survey questions are ordered according to hierarchical clustering of mean Likert scores for each group. (**B**) Bar plot showing ‘Positive Experience Scores’ for each group. Standard deviation error bars are shown. Two-sample t-tests were performed, comparing between groups, and Bonferroni correction was applied. Adjusted p-values are shown as significance stars; *ns* Not significant, **p < 0.01, ****p < 0.0001. Plots produced using ggplot2 in R.

Participants’ experiences of the different device types were qualitatively assessed with a Likert-style survey, which consisted of 16 questions scored on a five-point scale from strongly disagree through to strongly agree. Mean Likert scores for all questions (with strongly disagree scoring as 1, and strongly agree scoring as 5) were generated for participants grouped according to device type and vision type (with VI and sighted). Mean scores for the 16 questions were then clustered using hierarchical clustering to determine which questions were scored similarly (Supp. Fig. 2), and these data were plotted as a heatmap (Fig. 6A).

For participants with VI, Shape was generally scored more favourably than Vibration (Fig. 6A). In particular, participants with VI scored Shape towards agree / strongly agree and Vibration towards disagree, for three questions that asked whether the intended direction to a target was easy to interpret while using the device (Fig. 6A). For sighted participants, Vibration was scored very poorly, and considerably more so than for participants with VI, while Shape and sight were scored positively and similarly (Fig. 6A). This underscores the efficacy of the Shape device interface and suggests that vision capacity may determine how different digital navigation interfaces are perceived.

To quantify these data further, we designed a ‘Positive Experience Score’ (PES). The PES consisted of 6 questions from the Likert survey that were deemed to indicate a positive user experience (and were scored from 1 to 5 for strongly disagree to strongly agree), and 5 questions from the Likert survey that were deemed to indicate a negative user experience (and were scored from 5 to 1 for strongly disagree to strongly agree) (Supp. Table 1). Mean PES scores for participants were grouped together according to device and vision type (with VI and sighted) and were visualised as a bar plot in Fig. 6B. Participants with VI and sighted participants scored Shape significantly more positively than Vibration according to the PES, and as described above, Vibration was scored comparably poorly by sighted participants (Fig. 6B). Moreover, Shape was scored more positively (as actual mean PES values) than sight by both participants with VI and sighted participants, and there was found to be no statistically significant difference between Shape and sight PES values for sighted participants (Fig. 6B).

Within the survey, participants had the opportunity to write freehand comments to describe their user experiences. These are useful for clarifying a participant’s experience, as well as providing extra information not covered by the questions in the survey. Some examples taken from individuals with VI for Vibration were the following:

“Mainly using the noise [of the device] to locate the targets”.

“Frustrating; arm got tired [and developed] an RSI-like strain from using it for long durations”.

For Shape, a range of comments were received including some suggestions for device and trial design improvement. However, some individuals with VI did also comment positively about the device, and included the following:

“[The device was] fairly quick to learn, fairly sensitive, and not tiring”.

These comments support the Likert data and are useful reflections when considering how to optimise and modify Shape to be a comprehensive navigation device for individuals with VI.

## Discussion

Findings presented here build on our previous work and expertise in shape-changing haptic interfaces, however this time with a specific focus for individuals with VI. We have shown that our device, Shape, outperforms Vibration in speed (Fig. 3) and efficiency (Fig. 4) when locating 3D virtual targets, and in several instances, has been shown to be comparable to using natural vision. Moreover, participants generally scored their experience of Shape more positively than Vibration, and comparably to sight (Fig. 6).

This trial represents a first spatial guidance assessment of Shape in a controlled indoor situation. Moreover, several important aspects have been revealed. It’s been shown that the bending aspect and haptic design of Shape is intuitive to both individuals with VI and sighted persons and is effective at navigating individuals to highly variable targets. Indeed, this builds upon our previous demonstration of the S-BAN device^4^, and supports the use of these types of device interface for navigational purposes. Moreover, our data suggest, as shown in other studies^24–28^, that vibration is an ineffective interface for navigational guidance, which is frequently scored negatively by users for a variety of reasons.

Looking ahead, we aim to assess how Shape performs at navigating individuals (both with VI and sighted) in a pedestrian-style trial setup. Here, participants would be guided to walk to a target in a controlled situation, while avoiding obstacles located on the route (such as soft, inflatable cones). This type of trial will enable us to assess the functionality of Shape in a scenario that more closely reflects the outside environment. Indeed, our study’s findings suggest that Shape could be highly effective in this type of setup. Moreover, this scenario may allow for combining Shape with other kinds of technology, such as teleassistance tools, so that the tested device accurately resembles a tool that will be useful in a real-world situation.

A key aspect of this study is the evidence provided for shape-changing haptic interfaces as potent tools for representing spatial information, which may be used in future navigation systems. Our previous work has shown these interfaces to be useful in a variety of navigational and perceptual studies^3,4^; however, this study provides new and useful evidence specifically for VI navigation. This is particularly important given the challenges that individuals with VI face while navigating their outside environments and the current lack of digital alternatives to traditional navigational aids. We now aim to build on our findings to optimise, refine, and advance Shape to be the market-first shape-changing haptic navigational tool for individuals with VI.

## Limitations and future work

The work presented in this manuscript aimed to evaluate the potential of the Shape device as an interface for communicating spatial guidance. To ensure that the collected high-resolution user-motion data was suitable for reliable comparison between the three modalities (Shape, Vibration and Sight), we designed our experiment to be a controlled simplification of real-world navigation. This approach follows typical scientific design, enabling the isolation of important variables by reducing or eliminating variation in other factors. As a result, there are several aspects of our system that do behave as would be necessary in a realistic VI pedestrian guidance scenario. These limitations will be discussed in the following subsections.

### Perception system limitations

Our study currently involves the sequential presentation of 60 invisible targets, that exist only in a VR system. By taking this approach we did not need to rely on a perceptual system to identify and locate physical targets.

Such a system could be a device-mounted camera that identifies the location of objects or fiducial markers placed around our environment. Indeed, such vision systems are commonly used in robotics applications and would allow direct extension of Shape into realistic environments. The images from the camera could be processed with machine learning (ML) to track useful visual features in cluttered environments. However, vision systems inherently feature noise due to lens distortion, motion blur and variations in lighting. Additional latency also occurs due to ML-based image processing. Such noise leads to less reliable target localisation and inconsistent study conditions between participants, as was determined in our past work on outdoor pedestrian navigation using GPS^25^. Furthermore, it would be difficult to fit many physical targets into the limited workspace of a study environment without introducing further errors from the close proximity of neighbouring targets.

By using the *HTC Vive Pro* virtual reality system for localisation, we were able to track the user and device at 100 Hz with a precision of less than 0.1 mm. Such resolution far exceeds what would be possible with a vision-based perception system. Despite this, due to the non-portable nature of VR tracking systems, we are indeed planning on using a device-mounted vision system in our future work on real-world navigation experiments.

### Static vs. mobile studies

In the presented study, participants remained relatively stationary in a 2 × 2 m environment while virtual targets appeared around them. This was a simplification of the guidance-while-walking scenarios we envision for the future of Shape. Again, this decision was made to reduce environmental variables and focus only on collecting high-resolution motion data related to pointing at targets. Our past outdoor navigation work highlighted to us that significant variations in study conditions occur across participants due to weather and other pedestrians^25^.

Now that we have collected data on Shape in controlled conditions, we are preparing for outdoor navigation experiments in our future work. These studies are again intended to involve participants with VI, who will use Shape alongside their current mobility aid (a white cane or guide dog) to follow a pre-defined path (made of multiple targets) through an urban environment to a destination. We are particularly interested in whether the guidance provided by Shape will compliment current mobility aids or cause conflicts related to attention and cognitive load.

### Multiple targets

While the virtual targets in this study are presented individually and sequentially (to generate directly comparable motion data), in real-world guidance scenarios there is often likely to be multiple potential target options in one cluttered scene. Some additional interface will clearly be required to present the available target options to the user and allow them to choose the desired option, which Shape will then guide them too. The optimal method for such high-level contextual HCI (which is likely to involve generated speech) is highly important, but beyond the scope of our current investigations into communicating pure spatial information.

### Combining shape and vibration

Our study compares user behaviour when guided by the haptic modalities of shape-change and vibration, determining that shape is superior for spatial guidance. However, in a practical system it is likely that both modalities will be employed, with each leveraged for their distinct perceptual properties. It was previously mentioned that vibration is excellent at providing attention-grabbing alerts, which can become problematic when activated too often. Therefore, we envision a hybrid system where shape feedback is mostly used for general mobility guidance (i.e. following a trajectory through an urban environment), while vibration is used to indicate approaching hazards that require immediate attention. The spatial location of these hazards could then be further indicated by Shape.

An alternative use of vibration could be to alert users to uncertainty in the device’s perceptual system. This type of feedback is analogous to the visual ‘circle of uncertainty’ displayed in Google Maps, which indicates error magnitude without interrupting guidance^31^.

### DOF of shape and vibration modalities

In this work, we have comparatively evaluated 2-DOF shape-change feedback, 1-DOF vibrational feedback and natural vision. Our decision to involve 1-DOF vibrational feedback was based on current commercially available navigation interfaces for persons with VI, as opposed to multi-actuator vibrational systems from research literature (e.g.^19–23^.). Current commercial interfaces include smartphones/smartwatches (with apps such as HapticNav or GoodMaps), smart canes (e.g. WeWalk, Gosense) and wrist bands (e.g. Sunu and WayBand). These devices all feature a single vibration actuator (either an ERM or LRA).

As such, modality choice was not based explicitly on DOF, which is why natural vision was included. Indeed, the DOF of sight might be considered as 3-DOF for the virtual spherical targets, based on translational Euclidean coordinates.

A further factor that influenced our study design was keeping the form factor of the user interface consistent across all modalities, to remove confounding factors from certain devices being more ergonomic than others. It should be noted that much successful past work on multi-DOF vibrational feedback has focused on wrist^19,21^ or ankle^22,23^ worn wearables rather than handheld systems, which provide quite different qualitative experiences.

## Methods

### Device hardware

The goal of the Shape interface is to communicate 3D vectors with fidelity equivalent to natural sight. The device contains a two degree of freedom joint to allow the head to be pitched and yawed relative to the handle. This generalises the pointing motions of the previous (2-DOF) S-BAN device, which provided *distance* and *heading* to targets, but within the limitations of a 2D plane^4^. In this study, the targets have a fixed distance but are able to move out of plane, opening up future work of real-world target localisation applications for persons with VI (Fig. 2). Shape also has ergonomic benefits compared to the S-BAN. In addition, the Shape device contains a Linear Resonant Actuator (LRA) to provide an alternative vibrotactile modality. This allows all experiments to be carried out with the same device reducing the confounding effects of ergonomics and weight.

The device is designed to be used with an “inline” grip, like a flashlight. The diameter of the head and body is a maximum of 32 mm, this maximises grip for a wide range of hand sizes and minimises strain when holding the device for prolonged periods. Despite being used for over 2 h by elderly participants, no complaints were received related to forearm fatigue.

The cross-section of the device is square with rounded corners, providing flat surfaces to allow better differentiation of alignment of the head and handle. In addition, located on the head are “tactile recesses” to encourage index and thumb finger placement. However, it was observed by several users that the flat sides of the device allowed a similar grip to a white cane, where the index finger is laid along the axis of the cane. This familiar geometry appeared to improve the confidence of the VI participants who observed it.

### Shape hardware

The 2 degree of freedom joint is driven by a tendon mechanism eliminating all actuators from the body of the device, the servos (Dynamixel XC330- M181-T) are placed externally to allow freedom in the ergonomic design of the handle and head (Fig. 1).

The handle is linked to the head via a flexible spline. This bends while maintaining a fixed length and preventing rotation of the head, limiting it to two degrees of freedom The head is controlled by 3 tendons (made of multi-core steel rope) making the linkage over-constrained, this eliminates the need for a separate tensioning mechanism. The servos applied a minimum of 0.05Nm of torque to ensure the mechanism is always tightly tensioned. The three servos provided a maximum of over 0.6Nm of torque each and allowed the full range of motion (± 50 degrees) to be traversed in 55 ms. The resultant output torque on the head joint is approximately 0.66Nm, which translates to 18N of pushing force on the thumb indent located in the middle of the top face of the head. This combination of high torque and fast motion allows the device to provide compelling feedback.

The functional components of the device were constructed from either milled aluminium or SLS printed nylon. This provided sufficiently high tolerances that measured spline bend accuracy was only ± 1 degree from the kinematically calculated bend angle. The mechanism was run in a bench test for 12 h continuously to test reliability. The primary failure point of the mechanism was the tendon attachment to the pulleys, this involved a tight (> 90 degree) bend, which caused the tendon to fray over time. This failure was observed twice during the length of the entire research project. A duplicate reserve device was built in case of such failure during experiments, but this did not occur.

### Vibration hardware

To provide the vibration modality a Linear Resonant Actuator (LRA) is contained within the centre of the handle. The position in the centre of the grip maximises tactile sensation by minimising the damping of the case. An LRA was chosen over an Eccentric Rotating Mass (ERM) as LRAs are found widely in contemporary consumer haptic devices, including iPhones since 2015. The Foster Electric 646752 vibration actuator is used in our device, which is the same unit as inside a Sony PS5 controller. The unit provides a maximum acceleration of 3.5G per pulse (https://www.foster-electric.com/products/vibration_actuator/index.html), approximately 10 times more than a generic smartphone LRA (https://nfpshop.com/product/x-axis-rectangular-linear-resonant-actuators-model-nfp-elv451230). For continuous excitation, LRAs are more efficient, providing twice the output force for the same input power while also having a much faster response time compared to ERMs^32^.

The input signal amplitude is independent of its frequency for an LRA. This allows more complex driving waveforms not possible on an ERM. The driving strategy uses a waveform with a period of 0.01 ms, this may be pulsed faster or slower to increase the perceived vibration frequency without a change in the magnitude. This includes patterns of high-intensity pulses.

### Virtual reality system

The device is controlled by a *HTC Vive Pro* virtual reality system. This uses 4 base stations to monitor the 6DOF pose of two trackers and the headset at a frequency over 100 Hz with a precision of less than 0.1 mm^33^. The device has an HTC *tracker* mounted on a boom (Fig. 1), facilitating an ergonomic grip on the device and permitting the use of a rigid body transform to estimate the position of the handle. A second tracker is mounted on the participant, this tracks their centre of mass and is used to determine target position. The electronics to power and control the device are contained in a rucksack worn by the participant, and the next generation of devices contain this internally, making the interface pocket sized.

The targets for all trials are virtual spheres, that exist only in the virtual reality environment and have no physical equivalence within the study space. The only time the targets are visible is during the Sighted Task, at which time only the current target is displayed to the participant via the HTC Vive virtual reality headset (see Supp. Fig. 3). Participants do not wear a headset nor blindfold for the Shape or Vibration tasks.

### Tilt compensation

The position of the target is estimated relative to the device’s fixed reference frame. This tilt-compensates the device at all times, ensuring it communicates irrespective of roll about its axis. The supplementary video shows the Shape modality demonstrating tilt compensation and the range of motion, though tilt compensation was also applied to the vibration mode.

### Device behaviour

The device was given three modes to allow evaluation of Shape, Vibration and Sight guidance.

### Shape behaviour

The device bends the head of the device to point towards its target. The goal of the user is to straighten the device, this provides a similar sensation to gripping a handrail, the bending direction is towards the desired target. Once the user was pointing towards the target the LRA was used to provide a continuous thumping sensation. This multi modal haptic approach using vibration for an alert was put forward by Spiers and Dollar^3,26^.

### Vibration behaviour

The LRA was used to provide a Vibration of increasing frequency from 1 to 15 Hz to indicate increasing accuracy as the device was pointed towards the target. This 1D vibratory sensation was provided with constant magnitude ensuring the sensation was always equally detectable.

Numbness or discomfort caused by exposure to vibration is well documented^26,34–36^. Due to the relatively long task duration of our study (> 30 min for some participants) we used a maximum LRA vibration frequency which was intended to reduce the occurrence of numbness or fatigue (15 Hz). This was selected based pilot testing, where a range of frequencies were trialled with the LRA. 1 Hz was chosen for the lower bound. We received no complaints of numbness, tingling or discomfort from participants.

The relatively low frequency of our vibration stimulus suits Slowly Adapting (SA) mechanoreceptors: They respond to sustained pressure and low-frequency vibrations, continuing to fire at a relatively constant rate throughout the duration of a stimulus. Rapidly Adapting (RA) mechanoreceptors are more sensitive to higher frequencies but can respond to low-frequency stimuli as discrete events, they respond vigorously at the onset and offset of a stimulus but quickly decrease their firing rate if the stimulus is maintained^35,37^**.** Our frequency targeted a range which would to be interpreted as individual taps to stimulate SA mechanoreceptors and repeatedly activate the RA mechanoreceptors rather than continuously stimulate them, generating a continuous firing rate without fatigue.

Once pointed at the target the rhythm of the vibration would change to a “double-time" or “heartbeat” type pattern. Haptic rhythm perception is expected to perform similarly to auditory equivalents, making this easily detectable, even to those with hearing impairment^38^.

A multi vibration approach was considered, however this does not represent the vibration guidance interfaces currently available to VI individuals. Products which use a single vibration source include all smartphones and most VI specific navigation devices such as WeWalk (https://wewalk.io/en/), Gosense Rango (https://www.gosense.com/rango-smart-white-cane/), Sunnu Band (https://sunu.io/), and WayBand (https://www.wear.works/).

### Sight

The sighted tasks were performed using the VR Headset, allowing participants to see the virtual targets and a model of the device, see Supp. Fig. 3. The inclusion of a device model provided feedback such that the users could judge when they perceived themselves to be on target. This represents the sensory situation for sighted people who do not use any vision aids beyond glasses. The headset includes sufficient space such that participants could wear glasses during the test.

### Trial design

Virtual targets were generated on a spherical frustrum of radius 3 m centred on the chest tracker worn by the participant. An image of the frustrum is included in Fig. 2, the shape ensured all the targets started 3 m from the participant and were not directly above or below them. The set 3 m distance ensured the competing effects on accuracy of translating and rotating the device where both present when searching for the target as they would be when attempting to find stationary objects in a practical application. Centring the frustrum on the chest tracker allowed the participant to drift with the 2 × 2 m test area while maintaining constant distance to the targets at the start of each task. The zenith angle from one point to the next was normally distributed (mean 90) so as not to give direct advantage/disadvantage to natural sight.

As described in Fig. 2, each participant was equipped with the hardware device, on which an HTC Vive Tracker was mounted and a rucksack containing the necessary electronics to power the device. They were then provided with a short familiarisation and training session consisting of: an unpowered demonstration of device ergonomics, a powered demonstration tracking a physical target (another HTC Vive tracker), a training period with virtual targets. The test period with the physical target allowed the users to understand the behaviour of the device in response to target movement using proprioception. As sighted participants could see the physical tracker, allowing individuals to feel the device reaction made the training agnostic of sight loss severity. This training approach was repeated for the vibration and sighted experiments.

While standing within a clear 2 m × 2 m area, the participant was tasked with pointing the handheld device at a series of 60 virtual targets located in 3D space around them. For Shape and Vibration, the device provided haptic feedback to guide the user to point at the target. For Sighted tasks, the users were given a VR headset to allow them to see the targets and the device to point at the targets. There was no difference in trial conditions between Sighted and VI participants for the Vibration and Shape tasks. Participants were requested to look straight ahead and avoid looking directly at the device, this instruction was repeated during the experiments if participants appeared to be looking at the device. This instruction applied also to participants with VI, in case of residual vision. The device provided no haptic feedback for sighted tasks. Following each session, participants were asked to complete a Likert survey questionnaire to describe and score their experience. Data was collected in compliance with the Data Protection Act 2018 and General Data Protection Regulations (Europe).

### Ethical approval

The study was approved by the internal head of department and received a favourable opinion and approval from the Science, Engineering and Technology Research Ethics Committee (SETREC number: 6356785). All experiments were performed in accordance with relevant guidelines and regulations. Informed consent was obtained from all participants.

### Participant recruitment

Participants were recruited to the study through MakeSense Technology and the Blind Veterans UK network. Inclusion criteria for the study stipulated that participants must be at least 18 years old; must have basic computer literacy, such as being able to open and read documents, and open, write, and send emails; and must be able to speak English to a good standard and be able to understand study instructions. Exclusion criteria for the study stipulated that participants must not have any motion or sensory impairments in their hands or arms that may affect how the device is held and perceived. VI participants were defined as being legally blind in the United Kingdom. Typical cane and guide dog use by participant were not recorded as the exercise did not directly involve navigation. Signed or verbal-recorded consent to participate in the study was obtained from all participants after they had been provided with a full explanation of the study, an information leaflet had been offered, and time allowed for consideration.

### Data and statistical analysis

Data were processed in R (v.4.3.1), MATLAB (R2023b) and Python (3.10), and all code are reproducible and can be made available. Data glitches and incomplete tasks were first removed from the data, and statistical outliers were detected and removed using the interquartile range method (IQR; > 1.5*IQR above Quartile 3, and < 1.5*IQR below Quartile 1); of note, this was performed independently for time taken to complete tasks and for angular distance per task. Processed data were used for downstream plotting.

To assess average time taken to complete tasks, data were visualised as boxplots using ggplot2 (v.3.4.3), two-sample Welch’s *t*-tests with Bonferroni correction were applied to data using rstatix (v.0.7.2), and adjusted *p*-values were displayed as significance stars on plots.

To assess rotation efficiency, the angular distance was measured using the *dist* function in MATLAB between starting and ending quaternion rotations for each task (optimal route), and then between each collected quaternion rotation from starting through to ending rotations for each task (actual route taken). Distances were outputted in degrees. Device positions were kept constant throughout post-processing, meaning that translation was not included in the metric. Rotation efficiency was then calculated using the equation: (Optimal Route / Actual Route)*100. Efficiency values were visualised as boxplots, two-sample Welch’s *t*-tests with Bonferroni correction were applied to data, and adjusted *p*-values were displayed as significance stars on plots.

Heatmaps were generated from mean Likert scores using *ggplot2*, and survey questions were ordered according to hierarchical clustering of the data using the stats package in base R. The ‘Positive Experience Score’ was generated from 6 questions considered to indicate a positive user experience (scored 1 to 5 from strongly disagree through to strongly agree), and 5 questions considered to indicate a negative user experience (scored 5 to 1 from strongly disagree through to strongly agree) (Suppl. Table 1). Mean scores from these data were calculated, and participants were grouped according to device and vision type. Due to the small sample size, a more extensive analysis using Rasch Modelling or Item Response Theory was not applicable^39^.

Quaternion rotation values for individual tasks were plotted sequentially to create animations using the *poseplot* function in MATLAB. A mesh STL file was created using Solid Works and was used to represent the device. Example animations may be viewed in Supp. Fig. 1.

## Supplementary Information

Supplementary Video.

Supplementary Figure 2.

Supplementary Figure 3.

Supplementary Legends.

Supplementary Table 1.

## Data Availability

The datasets generated and analysed during the current study are available in the ‘Shape_Phase1_Data’ repository, which is hosted at: https://www.dropbox.com/scl/fo/l03hszwgzq7mltfml5gl8/h?rlkey=4n67472uctexzoc3visiu1une&dl=0
