# Supplementary figures and images for: A shape-changing haptic navigation interface for vision impairment
Source: Sci Rep. 2024 Dec 10;14:29223. doi: 10.1038/s41598-024-79845-7 (PMC11632113; doi:10.1038/s41598-024-79845-7)

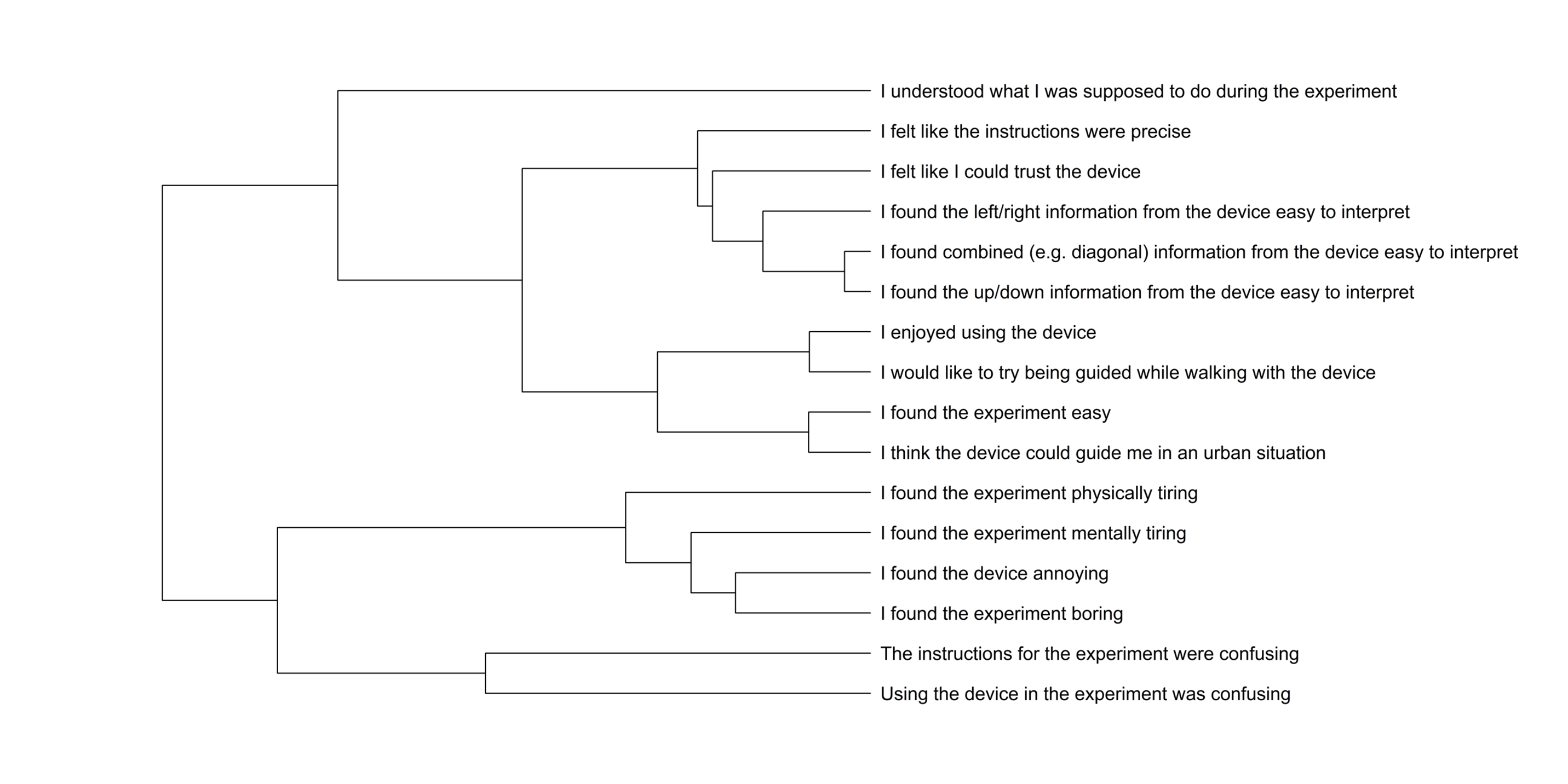

Supplement: Supplementary file 2 — Supplementary Figure 2. [file 41598_2024_79845_MOESM2_ESM.png]

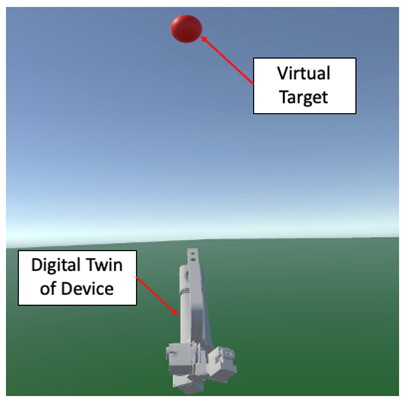

Supplement: Supplementary file 3 — Supplementary Figure 3. [file 41598_2024_79845_MOESM3_ESM.png]
