# Supplementary material for: A shape-changing haptic navigation interface for vision impairment
Source: Sci Rep. 2024 Dec 10;14:29223. doi: 10.1038/s41598-024-79845-7 (PMC11632113; doi:10.1038/s41598-024-79845-7)
Supplement: Supplementary file 4 — Supplementary Legends. [file 41598_2024_79845_MOESM4_ESM.docx]

Supplementary Figure 1. Representative animations for locating a study target for a participants with VI and with sight.

Animations are for participants P6 (with VI) and S4 (sighted), and how these participants located target number 4 in the study with all device types (as depicted with time series graphs in Figure 5). Animations were generated using MATLAB.

Supplementary Figure 2. Hierarchical clustering of mean Likert survey scores.

Hierarchical clustering was performed on mean Likert scores to reveal trends and patterns within and between survey questions. The clustering order was used to fit the heatmap shown in Fig. 6A.

Supplementary Figure 3. The visual experience of sighted task is viewed through a HTC Vive virtual reality headset. The view involved a 3D digital twin of the Shape device (which is mapped to the motion of the handheld device in real time), the current virtual target (a red sphere) and a simple background consisting of a green floor and blue sky, with distant horizon. As in all tasks, the participant’s goal was to point the device at the virtual target.
