# Supplementary material for: A shape-changing haptic navigation interface for vision impairment
Source: Sci Rep. 2024 Dec 10;14:29223. doi: 10.1038/s41598-024-79845-7 (PMC11632113; doi:10.1038/s41598-024-79845-7)
Supplement: Supplementary file 5 — Supplementary Table 1. [file 41598_2024_79845_MOESM5_ESM.pptx]

## Slide 1
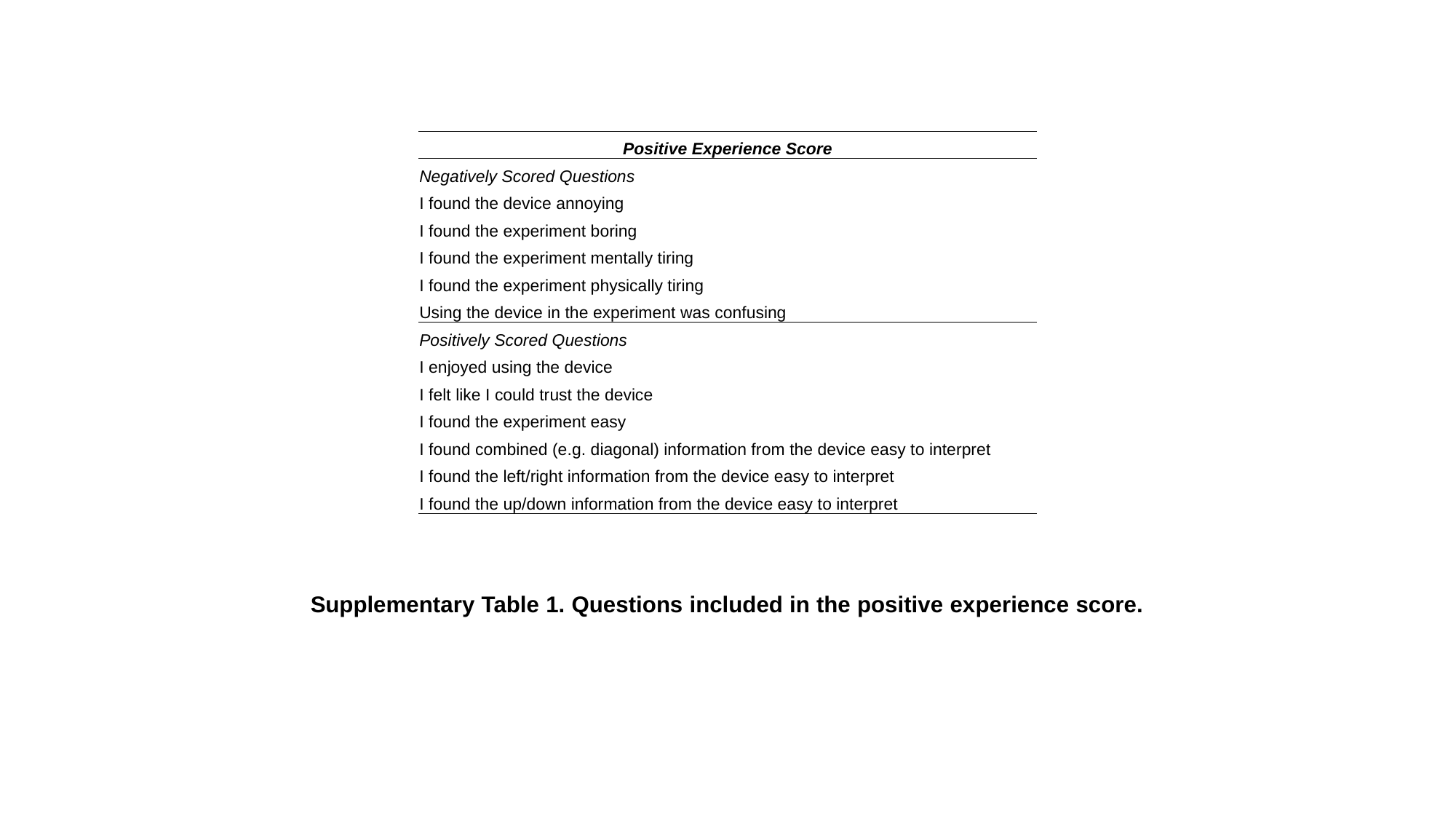

| Positive Experience Score |
| --- |
| Negatively Scored Questions |
| I found the device annoying |
| I found the experiment boring |
| I found the experiment mentally tiring |
| I found the experiment physically tiring |
| Using the device in the experiment was confusing |
| Positively Scored Questions |
| I enjoyed using the device |
| I felt like I could trust the device |
| I found the experiment easy |
| I found combined (e.g. diagonal) information from the device easy to interpret |
| I found the left/right information from the device easy to interpret |
| I found the up/down information from the device easy to interpret |
Supplementary Table 1. Questions included in the positive experience score.
